# Bile Acid Detection Techniques and Bile Acid-Related Diseases

**DOI:** 10.3389/fphys.2022.826740

**Published:** 2022-03-16

**Authors:** Xiang Zhao, Zitian Liu, Fuyun Sun, Lunjin Yao, Guangwei Yang, Kexin Wang

**Affiliations:** ^1^Cheeloo College of Medicine, Shandong University, Jinan, China; ^2^Department of General Surgery, Qilu Hospital of Shandong University, Jinan, China

**Keywords:** bile acid, detection techniques, related diseases, enzyme analysis, chromatography

## Abstract

Bile acid is a derivative of cholinergic acid (steroidal parent nucleus) that plays an important role in digestion, absorption, and metabolism. In recent years, bile acids have been identified as signaling molecules that regulate self-metabolism, lipid metabolism, energy balance, and glucose metabolism. The detection of fine changes in bile acids caused by metabolism, disease, or individual differences has become a research hotspot. At present, there are many related techniques, such as enzyme analysis, immunoassays, and chromatography, that are used for bile acid detection. These methods have been applied in clinical practice and laboratory research to varying degrees. However, mainstream detection technology is constantly updated and replaced with the passage of time, proffering new detection technologies. Previously, gas chromatography (GS) and gas chromatography-mass spectrometry (GC-MS) were the most commonly used for bile acid detection. In recent years, high-performance liquid chromatography-tandem mass spectrometry (HPLC-MS/MS) has developed rapidly and has gradually become the mainstream bile acid sample separation and detection technology. In this review, the basic principles, development and progress of technology, applicability, advantages, and disadvantages of various detection techniques are discussed and the changes in bile acids caused by related diseases are summarized.

## Introduction

Bile acids are a component of bile. They play a role in human metabolism by facilitating the digestion and absorption of lipids, maintaining the dissolved state of cholesterol in bile, and inhibiting its precipitation. In addition, bile acids can also act as signaling molecules to regulate self-metabolism, lipid metabolism, energy balance, and glucose metabolism by binding to the “membrane bile acid receptor”—Takeda G protein-coupled receptor 5 (TGR5) and nuclear hormone receptors, such as farnesoid X receptor (FXR) (Thomas et al., 2008).

Although there is little difference in the structure as well as physical and chemical properties of various bile acids, their functions are different. In experimental studies, the separation and detection of bile acids have been widely used, but they have not been popularized in clinical diagnosis. However, with the increasingly clear relationship between the occurrence of diseases and changes in bile acids, separation and detection of bile acids will play an increasingly important diagnostic value and guiding role in the future. Recently, detecting and separating bile acids accurately and quickly has become an important aspect of experimental studies. This review lists the methods used for bile acid detection according to the technical classification. The aim is to explore the basic principles, applicable conditions, advantages, and disadvantages of the various technologies, as well as the clinical application of bile acid detection in the diagnosis of diseases.

## Bile Acids Overview

### Structure and Classification of Bile Acids

Bile acids comprise a mixture of several compounds that are similar in shape, structure, and function. They can be divided into free and conjugated bile acids. The free bile acids mainly include cholic acid (CA), chenodeoxycholic acid (CDCA), deoxycholic acid (DCA), and a small amount of lithocholic acid (LCA). The 24 carboxyl groups of the above free bile acids can combine with glycine or taurine to form various conjugated bile acids, including glycocholic acid, taurocholic acid, glycochenodeoxycholic acid, and taurochenodeoxycholic acid (Table 1). In addition to aminoacyl amidation with glycine or taurine and sulfation, three glycosidic conjugation pathways have been established both *in vivo* and *in vitro* at the end of the last century: glucuronidation, glucosidation, and *N*-acetylglucosaminidation (Marschall et al., 1992), and that let us recognize more bile acid types in the body.

**TABLE 1:** Chemical structure of some common bile acids, including free bile acids, glycine/taurine-conjugated bile acids, and sulfated bile acids.

Abbreviation	Compound	R1	R2	R3	R4	R5
CA	Cholic acid	α-OH	H	α-OH	OH	OH
CDCA	Chenodeoxycholic acid	α-OH	H	α-OH	H	OH
DCA	Deoxycholic acid	α-OH	H	H	OH	OH
7-oxo-DCA	7-oxo-deoxycholic acid	α-OH	H	=O	OH	OH
LCA	Lithocholic acid	α-OH	H	H	H	OH
12-oxo-LCA	12-oxo-lithocholic acid	α-OH	H	H	=O	OH
UDCA	Ursodeoxycholic acid	α-OH	H	β-OH	H	OH
α-MCA	α-muricholic acid	α-OH	β-OH	α-OH	H	OH
β-MCA	β-muricholic acid	α-OH	β-OH	β-OH	H	OH
ω-MCA	ω-muricholic acid	α-OH	α-OH	β-OH	H	OH
HCA	Hyocholic acid	α-OH	α-OH	α-OH	H	OH
HDCA	Hyodeoxycholic acid	α-OH	α-OH	H	H	OH
7-oxo-HDCA	7-oxo-hyodeoxycholic acid	α-OH	α-OH	=O	H	OH
DHCA	Dehydrocholic acid	=O	H	=O	=O	OH
MDCA	Murideoxycholic acid	α-OH	β-OH	H	H	OH
Unconjugated						OH
Glycine conjugates						NHCH2CO2H
Taurine conjugates						NHCH2CH2SO3H
Sulfated BAs	HSO4					

*α-MCA, β-MCA and ω-MCA do not have corresponding glycine conjugated bile acids, while bile acids with carboxyl groups (7-oxo-DCA, 12-oxo-LCA, 7-oxo-HDCA) do not have corresponding taurine and glycine conjugates.*

**FIGURE 1:**
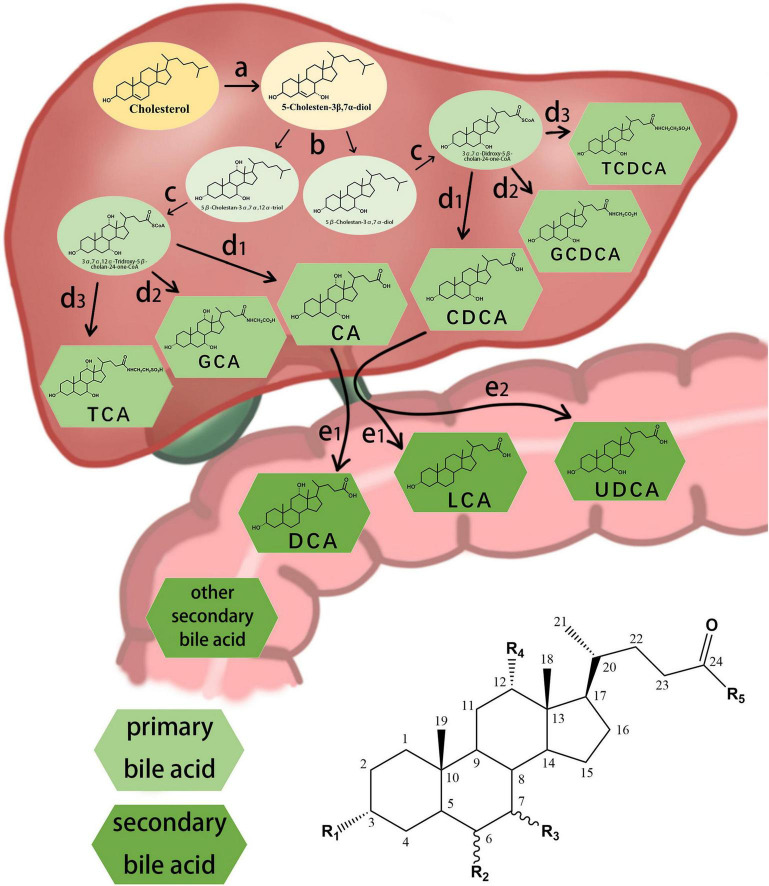
Chemical structure of some common bile acids and the synthesis primary bile acid and generation of secondary bile acid. (a) initiation of synthesis by 7-hydroxylation of sterol precursors, (b) further modification of the ring structures, (c) oxidation and shortening of the side chain, (d1) hydrolyzation of the bile acids, (d2) conjugation of the bile acids with glycine, (d3) conjugation of the bile acids with taurine, (e1) dehydroxylation of the bile acids, (e2) conversion of chenodesoxycholic acid acid 7α-hydroxyl to 7β-hydroxyl.

The different structures of bile acids are mainly reflected in the occurrence of hydroxyl or carbonyl groups on the carbon atoms at specific positions. The basic structure of most bile acids is shown in Figure 1. The molecular structures of some common bile acids, based on replacing the functional groups shown in Figure 1 are listed in Table 1 (Tagliacozzi et al., 2003; Mano et al., 2006; Gowda, 2011; Jantti et al., 2014; Liu et al., 2018; Dutta et al., 2019).

Bile acids can be divided into primary and secondary bile acids based on their sources. Primary bile acids are synthesized by the liver and stored in the gallbladder as an important component of bile. They mainly include CA, CDCA, and their combined products with glycine and taurine.

### Bile Acid Synthesis and Enterohepatic Circulation

In the classical pathway, primary bile acids are synthesized in four steps: (a) initiation of synthesis by 7-hydroxylation of sterol precursors, (b) further modification of the ring structures, (c) oxidation and shortening of the side chain, and (d) conjugation of the bile acids with an amino acid (Russell, 2003). Secondary bile acids are synthesized by deconjugation, dehydrogenation, and dehydroxylation reactions of the gut microbiota (Figure 1; Chiang, 2013; Winston and Theriot, 2020). The gut microbial community, owing to its ability to produce bile acid metabolites different from those produced by the liver, may be considered an “endocrine organ” with the potential to alter host physiology, perhaps to benefit the parasitic gut microbiome (Ridlon and Bajaj, 2015).

Bile acids circulate in the digestive tract and internal circulation through enterohepatic circulation. Approximately 95% of bile acids that enter the digestive tract can be reabsorbed, and the remainder is excreted in the stool. Bile acids can be reabsorbed in two ways: Conjugated bile acids are actively reabsorbed in the ileum by bile acid transporters, while free bile acids are passively reabsorbed in all parts of the small intestine and large intestine. Reabsorbed bile acids re-enter the liver via the portal vein. In hepatocytes, free bile acids are reconverted into conjugated bile acids, which are secreted into the intestine with bile along with reabsorbed and newly synthesized conjugated bile acids. This cycle is called the enterohepatic circulation of bile acids. Small amounts of bile acids that are not acquired by liver cells enter the blood circulation through the hepatic vein. Thus, in addition to the liver and bile, bile acids are also present in the blood, urine, stool, amniotic fluid (Ushijima et al., 2001), sputum (Higashi et al., 2010; Durc et al., 2020), pleural effusion (Che et al., 2018), and bronchoalveolar lavage fluid (Bessonneau et al., 2014). The first three are often used as typical samples for bile acid detection.

### Relationship Between Bile Acids and Diseases

Because of the close relationship between bile acids and metabolism, the concentration of bile acids in each component also changes during diseases, such as hepatobiliary disease (Li et al., 2017; Jia et al., 2018), gastrointestinal disease (Duboc et al., 2013; Lee et al., 2020), metabolic disease (Wu et al., 2015; Lackey and Olefsky, 2016), and nervous system disease (Grant and DeMorrow, 2020). 3α-Hydroxysteroid dehydrogenase (HSD) enzyme assays have been used to detect total bile acid (TBA) concentration in clinical practice to diagnose the occurrence of diseases. However, most diseases only lead to an increase or decrease in the concentration of one or several bile acids, but not TBA. For example, Zheng et al. (2021) found an association between hyocholic acid (HCA) and metabolic disorders, such as obesity and diabetes. The feasibility of using HCA to assess the risk of metabolic abnormalities in the future is further clarified. Thus, the separation of various bile acids and detection of their concentration changes in samples are conducive to scientific research, disease diagnosis, and even disease prediction, which is very important in the development of human medical and health undertakings. Therefore, the significance of bile acid detection needs to be evaluated.

## Bile Acid Detection Techniques Reported in Literature

Since the 1950s and the 1960s, there has been an increased interest in bile acid metabolism, and the related separation and detection technologies have emerged in an endless stream. Some of these methods have already been widely used in other detection fields and later applied to the detection of bile acids, such as spectrophotometry and early chromatography. In addition, after decades of research on bile acid metabolism, new separation and detection techniques have been established and quickly applied to the detection of bile acids.

The separation and detection methods of bile acids can be broadly divided into two categories: non-chromatographic and chromatographic. Non-chromatographic methods include enzyme analysis, radioimmunoassay (RIA), enzyme-linked immunosorbent assay (ELISA), nuclear magnetic resonance (NMR), and capillary electrophoresis (CE) (Table 2); among these, 3α-HSD enzyme analysis has been used in clinical diagnosis, and other methods are widely used in scientific research to varying degrees. Chromatography originated in the early twentieth century and developed rapidly after the 1950s. The basic principles underlying all kinds of chromatography are the same. Earlier, gas chromatography (GC) or gas chromatography-mass spectrometry (GC-MS) was the most commonly used bile acid detection method; in recent years, high-performance liquid chromatography-tandem mass spectrometry (HPLC-MS/MS) has developed rapidly and has gradually become the mainstream method for bile acid sample separation and detection. These methods have high sensitivity, good specificity, and low detection limit; however, they also have a few shortcomings.

**TABLE 2:** Advantages and disadvantages of the bile acid detection techniques.

Method		Advantages	Disadvantages
Enzyme analysis		Simple operation; low cost; applicable for clinical and scientific research to detect TBA, especially for clinical diagnosis	Only applicable for bile acids containing 3α-OH; high detection limit; failure to distinguish between the specific types of bile acids
RIA		Applicable for detection of trace bioactive substances at that time	Need of special equipment; radioactivity; cross-reactivity
ELISA		Small number of instruments; short analysis time; simple operation	Cross reaction; difficult to recover from serum; low accuracy
Spectrophotometry		Small number of instruments; short analysis time; low cost; simple operation process	Failure for the separation and detection of bile acids; low detection accuracy
NMR		Small sample size; simple sample pretreatment; capable of non-invasive	Relative low sensitivity compared to MS-based approaches
Chromatography	TLC	Simple operation process; low cost; good repeatability	Lack of direct quantification and low accuracy
	HPLC	High sensitive; simple to perform	Time-consuming; complex sample pretreatment
	SFC	Short detection time; not limited by the volatility and thermal instability of the compounds	Low reproducibility
	LC-MS	Short detection time; narrow peak width; low detection limit; high retention capacity; high signal-to-noise ratio	High cost
	GC-MS	Low detection limit; high retention capacity; high degree of separation	Complex sample preparation
CE		Good separation capacity and detection speed	Technically demanding; usually unreliable

To understand the practical application of bile acid detection technology in scientific research in recent years, we reviewed 105 articles reporting bile acid detection technology (the search was conducted on PubMed using the keyword “bile acid,” and the filter condition was “5 years.” Five hundred articles were selected, with an emphasis on those with an impact factor above 3). These studies are expected to aid the selection of appropriate bile acid detection technology.

**FIGURE 2:**
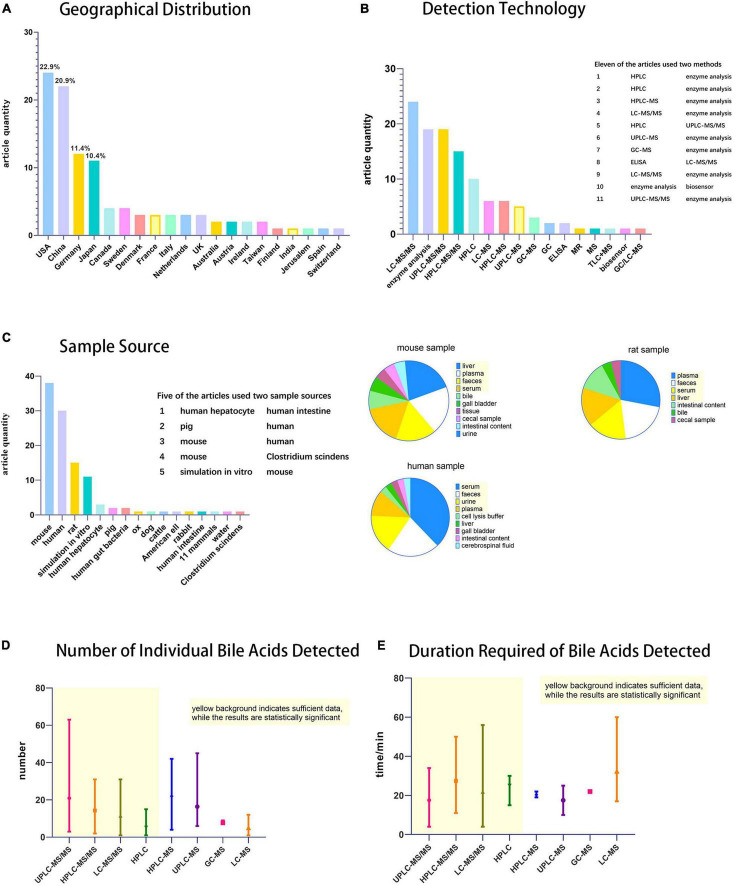
Statistical chart of bile acids detection techniques. **(A)** Histogram of geographical distribution of articles (*n* = 105) on bile acid detection technology, **(B)** Histogram of techniques used for bile acid detection. **(C)** Histogram of specimen sources and pie charts of specimen taken from mouse, human and rat. **(D)** Statistical chart of the number of individual bile acids detected and **(E)** duration required. Among Figures 2D,E, the part with yellow background indicates that the data is sufficient and therefore has statistical significance, while the rest has little statistical significance because of the small number of statistics.

First criteria was the geographical distribution of the detection technology (Figure 2A). Bile acid detection technology has been used in the United States, China, Germany, Japan, Canada, Sweden, and many other countries. Among them, the United States and China published the most relevant reports, with 24 and 22 articles, respectively.

Second criteria was the use of detection technology (Figure 2B). Many of the techniques mentioned in this review, such as RIA and CE, have not been used in scientific research. Most of these studies (79 in total) used LC-MS (LC includes HPLC and UPLC; MS includes MS and MS/MS), and GC-MS. Most MS detectors mentioned in specific experimental steps are tandem mass spectrometry. Kit/enzymatic tests were also frequently reported, but they only detect TBA as needed. It is a simple and convenient detection technique for experiments that do not require individual bile acid separation and detection. Among the statistical studies, 11 used two detection techniques, most of which are enzymatic methods combined with LC-MS, to separate and detect the concentration of individual bile acid components while measuring the TBA.

The third criteria was the specimens tested (Figure 2C). The sample sources were abundant. In addition to human specimens, there were various other types, such as experimental animals, *in vitro* simulations, cell culture, and even environmental bile acids can be detected. The most important specimens were mice, humans, and rats, accounting for 38, 30, and 15 articles, respectively. Five studies used two types of specimens according to experimental requirements. Because the experimental animals, such as rats and mice, can be subjected to anatomy experiments and extraction of the organs according to the rules and regulations, a wide range of specimens may be expected with an extensive sample detection range. Human specimens more accurately represent the metabolic changes in the body, not only in research experiments, but also in the clinic. Compared to experimental animals, human sample extraction is more likely to be less invasive or non-invasive, considering that the specimens do not usually include the liver, gut, or gallbladder.

Finally, the species and time required for bile acid detection were the two important aspects of our evaluation (Figures 2D,E). Among the four main detection techniques (UPLC-MS/MS, HPLC-MS/MS, LC-MS/MS, and HPLC), UPLC-MS/MS has the advantages of detecting more bile acid types and a shorter detection time, making it the preferred technology for the precise detection of bile acids.

## Bile Acid Detection Methods

### Enzyme Analysis

One of the chemical characteristics of bile acids is that they contain an α-hydroxyl group on the third carbon. 3α-HSD extracted from *Pseudomonas testosteroni* catalyzes the redox reaction of hydroxyl groups, and NAD^+^ is converted into NADH. The concentration of NADH is determined by spectrophotometry at 340 nm; thus, the concentration of bile acids in the sample is obtained indirectly. Based on this principle, Iwata and Yamasaki (1964) developed a method for measuring TBA (including free bile acids and bound bile acids) in samples. The method is relatively simple and fast, with a minimum detection limit of 5 μg.

In order to avoid the use of special instruments and relatively cumbersome pretreatments in the detection of TBA in plasma while ensuring satisfactory sensitivity, Mashige and his team improved the enzyme analysis method to measure bile acid concentrations in 1981 (Yamanaka et al., 1981). This method enabled the H of NADH generated in the first reaction to convert nitrotetrazolium blue into diformazan through diaphorase, and then detected the diformazan formed at 540 nm using a spectrophotometer. The minimum detection limit was 0.1 nmol. This method greatly improves the practicability of enzyme analysis in the detection of bile acid concentrations, is more suitable for general conventional laboratories, and has the characteristics for mechanization.

In addition to 3α-HSD, 7α-HSD can be used for bile acid detection. Roda and coworkers used a highly specific continuous flow method to detect the concentration of 7-α-hydroxyl bile acid by bioluminescence using nylon-immobilized bacterial enzymes (Bovara et al., 1984).

Enzyme analysis has been used in clinical and scientific research, especially for clinical diagnosis. Compared with this method, the other methods mentioned later have not been widely used in clinical practice. The total bile acid assay kit (total bile acid enzyme circulation method) has commercialized detection technology, facilitating the detection of TBA. The kit uses NADH, thio-NAD^+^, and bile acids to perform a redox cycle catalyzed by 3α-HSD, amplifying the signal and finally measuring the concentration of TBA by determining the absorption value of thio-NAD^+^ at 405/660 nm at 37°C using a spectrophotometer.

The disadvantages of enzyme analysis method are also particularly apparent. The method can only detect bile acids containing 3α hydroxyl groups and cannot be used if the hydroxyl groups are replaced by sulfuric or glucuronic groups. Moreover, the detection limit of this method is high, and it cannot accurately detect the bile acid content in some samples, such as saliva. In addition, enzyme analysis can only detect TBA and cannot distinguish between the specific types of bile acids. Therefore, the experimental data provided by this method only includes the total concentration, which is relatively limited. To obtain the respective concentrations of various bile acids, other methods that can separate and detect them should be selected.

### Immunoassay

#### Radioimmunoassay

Radioimmunoassay was established in 1959 by American scientists Professor Yalow and Berson as an *in vitro* method for ultrafine analysis. It resolved the issue of detection of trace bioactive substances, which was difficult to determine at that time, thus playing a significant role in promoting the development of modern medicine. RIA for bile acids in samples was first proposed by Simmonds et al. (1973). RIA utilizes the principle of specific binding between antigen and antibody and measures the concentration of the bound product by radioisotope labeling. However, because of the need for special equipment, radioactivity, and cross-reactivity, RIA has not been widely used, and few people have used this method to detect bile acid concentrations in recent years (Mäentausta and Jänne, 1979; Samuelson, 1980).

#### Enzyme-Linked Immunosorbent Assay

Since Engvall and Perlmann (1971) invented the ELISA technique in 1971, it has become an extremely important clinical and scientific assay. ELISA detects soluble antibodies or antigens in a sample. ELISA combines two basic principles: antigen–antibody specificity combined with enzymes and substrates for efficient catalysis. The results are based on the color changes of the enzyme substrate, using standard apparatus for quantitative analysis, with the sensitivity reaching the ng level. ELISA can be used to detect bile acid concentration in saliva samples that enzyme analysis cannot. Enzymes commonly used for marking include horseradish peroxidase and alkaline phosphatase. These enzymes are stable and can be preserved for a long time.

In 2017, Liu et al. (2017) used four different monoclonal antibodies to detect five bile acids [CA, DCA, CDCA, UDCA, and hyodeoxycholic acid (HDCA)] in plasma. Cui et al. (2017) established a biotinylated single-chain variable fragment-based ELISA method for the determination of glycocholic acid, and used a chicken single-chain variable fragment (scFv) antibody to detect glycocholic acid.

The method has the same sensitivity and accuracy as the RIA method, but does not require expensive equipment and is not radioactive. The experiment does not require the use of a large number of instruments and can effectively avoid the result error caused by cross contamination. The detection speed is faster than that of RIA or chromatography, and the operation is simple and easy to use; thus, it can be promoted in basic clinical detection. Its disadvantage is that the cross reaction between antigen and antibody occurs easily, which affects the accuracy of detection, false positives, and other false results (Dutta et al., 2019). Moreover, bile acids are difficult to recover from serum, and the recovery rate is always higher than the theoretical value measured by the standard curve without human serum.

### Spectrophotometry

Spectrophotometry is one of the most commonly used techniques in biochemical experiments. Its principle involves qualitative and quantitative analysis of the test substance by measuring the absorption of light at a specific wavelength or wavelength range. Most of the chemical bonds in bile acids are carbon–carbon single bonds without conjugated structures and luminescent groups; therefore, they cannot be measured directly by spectrophotometry. However, certain chemical reactions can cause the bile acids to exhibit an absorption peak at a certain wavelength or produce substances with an absorption peak at a certain wavelength. The principle is the same as that of the 3α-HSD enzyme analysis mentioned above. With regard to spectrophotometry, this review introduces other detection methods that use spectrophotometry.

Spectrophotometry eliminates the complex sample processing process and requires only a spectrometer, which is a common instrument in general QC laboratories. This method can be applied to a large number of samples because of its speed, low cost, and simple operation process. However, this method can only detect TBA or a certain type of bile acid at one time, and cannot be used for the separation and detection of bile acids, making it difficult to achieve accurate quantitative analysis of complex bile acid samples. Therefore, it is generally used for preliminary qualitative or simple quantitative analysis.

### Nuclear Magnetic Resonance

Nuclear magnetic resonance is a less commonly used method for the detection of bile acids. It is a spectroscopic technique that applies NMR phenomenon to the determination of the molecular structure. It can be used to detect and simultaneously quantify bile acid concentrations in the samples. In addition, NMR can provide detailed molecular structures and quantitative information. Duarte et al. (2009) first applied high-field NMR spectroscopy (800 MHz for 1 H observation) for the detection of human liver bile components (Duarte et al., 2009). NMR spectra of the entire extract showed the main bile acids (CA, DCA, and chenodesoxycholic acid), but failed to distinguish the conjugated bile acids of glycine and taurine. However, this method can be used to detect isomers of glycodeoxycholic acid and glycochenodeoxycholic acid, which cannot be detected by conventional liquid mass chromatography.

Owing to the high signal overlap of the NMR peaks of bile acids and the signal widening due to the low water solubility and aggregation of the main components of bile, NMR analysis is limited. This limitation was improved with the advent of two-dimensional (2D) NMR. 2D NMR overcomes the problem of overlapping resonances in the proton 1D NMR spectrum and is thus able to detect and align more metabolites than the 1D method.

Various other bile acid detection methods involve sample extraction, which is an invasive procedure. In recent years, NMR detection of bile acids has been of great significance in non-invasive *in vivo* examinations. Prescot et al. (2003) demonstrated the potential of monomeric magnetic resonance for non-invasive detection of the metabolic composition of bile in the human gallbladder. Unfortunately, the mass spectrum cannot detect molecules other than phospholipids, such as bile acids. More recently, Kunnecke et al. (2007) developed a non-invasive method for detecting bile composition in cynomolgus monkeys. Compared to the 1.5 T magnetic field strength used by Prescot et al. (2003) and Kunnecke et al. (2007) used a relatively high magnetic field (4.7 T) to detect and quantify different bile acid molecules, their taurine and glycine derivatives, and phospholipids for the first time.

The NMR method is relatively simple, requires a small sample size, has simple sample pretreatment steps, and avoids the detection errors caused by extraction, derivatization, hydrolysis, and purification. For example, the distribution of bile lipids in micromicelles and vesicles can be measured by NMR, which can provide information about the etiology of gallstone occurrence that is not possible with ordinary analytical methods, because other methods usually involve purification of the test substance.

### Chromatography

Chromatography is a common separation and analysis method that has a wide range of applications in analytical chemistry, organic chemistry, biochemistry, and other fields. This method is based on the slight difference in the distribution coefficient of the separated material between the two phases (stationary and mobile phases). This is a multistage separation technique. The principle is that when the two phases are in relative motion, the measured substance is repeatedly allocated between the two phases, further expanding the original small difference between the two and separating the components. According to the different stationary and mobile phases, chromatography can be divided into thin layer chromatography (TLC), HPLC, supercritical fluid chromatography (SFC), liquid chromatography-mass spectrometry (LC-MS), and GC-MS. The application of these five methods for bile acid sample detection is described below.

#### Thin Layer Chromatography

Thin layer chromatography is a chromatography separation technique that uses a support coated on the support plate as the stationary phase (in bile acid detection, it is commonly coated with silica) and an appropriate solvent as the mobile phase for the separation, identification, and quantification of mixed samples. It first appeared in the 1950s and was quickly widely used in the separation and detection of fatty acids, amino acids, nucleotides, and other substances (Stahl, 1956).

Thin layer chromatography can distinguish between free bile acids and glycine- and taurine-conjugated bile acids, but it cannot effectively detect and separate isomers of dihydroxyl-bound bile acids, such as DCA and CDCA. TLC has low specificity and cannot detect samples below 10 nmol per point; therefore, it is often used to detect bile or mixed standard solutions. Based on the basic TLC technique, two new techniques—2D TLC and reversed-phase TLC—have been developed (Momose et al., 1998; Onisor et al., 2010).

Since its conception and development, TLC technology has been characterized by its simplicity, low cost, and good repeatability. Owing to its lack of direct quantification and low accuracy, it widely used in routine clinical analysis and bile acid detection with low data requirements. When more accurate detection is required, the following chromatographic methods are selected (Poole, 2003).

#### High Performance Liquid Chromatography

High performance liquid chromatography is a widely used bile acid detection technique. For bile acid detection in samples, HPLC is performed with acetonitrile, ammonium formate, and formic acid solution as the mobile phase. Using a high-pressure infusion system, the pretreated samples and a mobile phase containing bile acids is pumped into a stationary phase chromatographic column (often as C18 reverse phase silica gel column), where each component is separated in the column and enters the detector for testing. An ultraviolet (UV) detector is commonly used for sample analysis. In HPLC, the modification of the chromatography method and the choice of detector and column depend on the sample type and purpose of the analysis. As the absorption of UV light by bile acids is very weak, derivations are often required prior to detection. However, detection of taurine and glycine combined with bile acid derivations are relatively simple compared with GC, and bile acids can be detected in the form of bile or other biological fluids.

In 1976, Shaw and Elliott (1976) first used HPLC to isolate and detect conjugated bile acids in human bile samples. Owing to the wide application of HPLC, many optimization techniques have been developed. Shi et al. (2015) reacted with 2-bromo-4′-nitroacetophenone with a carboxyl group on free bile acids under the catalysis of crown ether, resulting in a reaction product with a conjugated structure that could be detected sensitively by a UV detector at 263 nm. Onishi et al. (1982) used the post-column reaction technique of immobilized 3α-hydroxysteroid dehydrogenase to analyze bile acids using HPLC.

High performance liquid chromatography is highly sensitive and simple to perform; however, it is time-consuming and requires complex sample pretreatment, making it unsuitable for large-scale sample detection. Moreover, this method is limited by the matrix effect and the specificity of the detector coupled with the low absorption efficiency of bile acids for UV requires the selection of a lower wavelength UV light, which would aggravate the impact of this effect; thus, it is not suitable for the detection of complex samples, such as a variety of complex conjugated bile acids (Liu et al., 2018).

#### Supercritical Fluid Chromatography

Supercritical fluid refers to a fluid whose temperature and pressure are both higher than the critical point. Supercritical fluids have the dual characteristics of liquids and gases. SFC is similar to a separation and detection technology combined with HPLC and GC.

The mobile phase of SFC is carbon dioxide in the supercritical state. This is because the material has a relatively low critical temperature (31.1°C) and pressure (73.8 bar), is non-flammable, chemically inert, low cost, and has no waste problems (Scalia, 1995). In the supercritical state, carbon dioxide exhibits a solvation intensity close to that of a liquid, and can provide a lower viscosity and higher analyte diffusion coefficient, which is more efficient per unit time. The most interesting advantage is the flexibility of polarity. Polarity can be easily increased by adding a polar solvent, such as methanol. Therefore, the polarity of substances detected by SCF can be very wide (log Pow = –4.6–7.05) (Ishibashi et al., 2012). In 1991, Scalia and Games (1992) first used packed column SFC to detect free bile acids in preparations later applied this technique to detect conjugated bile acids in 1992 (Scalia and Games, 1993).

Owing to the small volume of gas generated by the mobile phase, SCF may be more easily linked to mass spectrometry than HPLC. In 2013, Taguchi et al. (2013), for the first time, used supercritical fluid chromatography-mass spectrometry to simultaneously detect 25 bile acids, including glycine- and taurine-conjugated bile acids, in rat serum samples within 13 min. Compared with HPLC, the detection time of SFC is shorter, the eluent has higher transparency at low UV wavelengths, and can use a sensitive and universal flame ionization detector. In contrast to GC, SFC is not limited by the volatility and thermal instability of the compounds. However, the SFC method has several disadvantages. The conjugated dihydroxyl isomers cannot be completely separated, and the reproducibility of the technique is lower than that of HPLC because the temperature and pressure of the critical fluid under the experimental conditions are not easily controlled. In addition, as the detection of bile acids by GC and HPLC has been relatively complete, SFC plays a less important role in the detection of bile acids.

#### Liquid Chromatography-Mass Spectrometry

**FIGURE 3:**
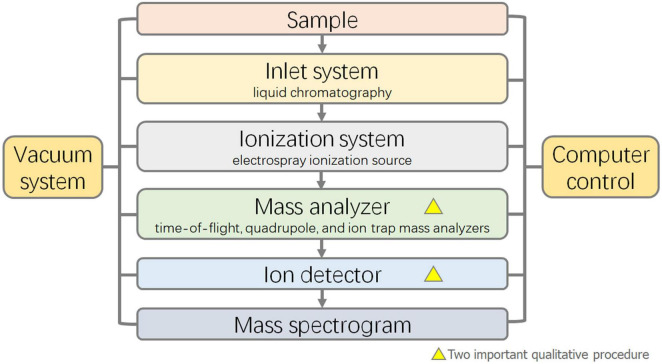
Flow diagram of Liquid Chromatograph-Mass Spectrometer (LC-MS).

Liquid chromatography-mass spectrometry is the most widely used technique for the detection of mixed bile acids because it can separate isomers of bile acids (Gómez et al., 2020). The separation ability was improved by using HPLC as a separation technique and mass spectrometry as a detection technique instead of a UV detector. The bile acids in the samples were separated by HPLC, and qualitative analysis was carried out by mass spectrometry using the ion mass-to-charge ratio. The principle and characteristics of HPLC are essentially the same as those mentioned above. In recent years, tandem mass spectrometry (MS/MS) has been commonly used for mass spectrometry analysis. Compared with first-order mass spectrometry, tandem mass spectrometry can selectively analyze partial fragments of the target, improving the sensitivity, accuracy, and signal-to-noise ratio. The core components of the mass spectrometer mainly include the ion source, mass analyzer, and ion detector (Yang et al., 1997). In the detection of bile acid samples, the majority of ion sources are electrospray ionization sources. Mass analyzers are commonly used in time-of-flight mass analyzers, quadrupole mass analyzers, and ion trap mass analyzers (Figure 3). While the full scan mode can be used for the identification and qualitative analysis of bile acids, selective ion monitoring (SIM) (Wegner et al., 2017), selective reaction monitoring (SRM) (Fu et al., 2020), and multiple reaction monitoring (MRM) (Tu et al., 2020) modes in tandem mass spectrometry are more helpful for the optimal selectivity and accurate quantification of bile acids (Dutta et al., 2019).

Liquid chromatography-mass spectrometry usually has high accuracy, with a detection limit usually of up to 0.1 ng/mL. The method is usually linear in the detection range of 0.01–1 μM. Biological samples used for bile acid detection mainly include serum, plasma, bile, tissue homogenate, saliva, adipose tissue, intestinal contents, urine, and feces. Before detection, simple sample pretreatment and the addition of an appropriate internal standard are needed for the quantitative detection of bile acids. The main purpose of pretreatment is to remove proteins, lipids, and inorganic salts. At present, protein precipitation, solid-phase extraction, and liquid–liquid extraction are used for sample treatment (Liu et al., 2018). The most commonly used method for plasma or serum is the protein precipitation method, which is simple in operation, requires less time, and is cost-effective. Acetonitrile or methanol is used as the precipitant, and the supernatant after high-speed centrifugation is used for detection. The internal standard method involves adding a pure bile acids of certain weight to the analysis sample as the internal standard. There are a variety of internal standards, such as deuterated bile acids (Alnouti et al., 2008; Bobeldijk et al., 2008; Higashi et al., 2010; Jantti et al., 2014), norDCA (Kakiyama et al., 2014), and dehydrocholic acid (Yang et al., 2017), among them, deuterated bile acids are the most common used. Then, chromatographic analysis of the sample containing the internal standard is carried out to determine the peak area and relative correction factor of the internal standard and the component to be measured, followed by quantitative detection of bile acids. To obtaining quantitative data on bile acids in samples, it is necessary to establish a standard curve. Bile acid calibration standard is dissolved in methanol. Normal samples identical to the samples to be tested are incubated with activated carbon to remove the original bile acids, and the blank sample solution is used as the biological matrix. The calibration standard curve can be obtained by adding a certain amount of dissolved calibration standard and testing it in the same way as the sample. The content of bile acids in samples can be quantitatively detected by combining the internal standard method with a standard curve (Krautbauer et al., 2016).

In addition to the conventional detection of free bile acids and glycine- and taurine-conjugated bile acids, this method has been widely applied to the detection of other bile acid derivatives. Ikegawa et al. (1999) developed a method for the simultaneous determination of 24-glucuronide bile acid in human urine by liquid chromatography-electrospray ionization-mass spectrometry. Bathena et al. (2013) developed a method for the simultaneous determination of bile acids and their sulfuric acid metabolites in human serum and urine by liquid chromatography-tandem mass spectrometry. Most LC-MS methods take longer than 20 min and exhibit moderate coverage of bile acid species (from 11 to 32 monitored species) as mentioned below.

Compared with GC, the pretreatment process of LC-MS is simple and less time-consuming. Compared with ordinary HPLC, the mass spectrometry detector of LC-MS is more expensive and complex. Although optimization of the mass spectrometry parameters requires certain experience, it has higher specificity, shorter detection time, and lower sample size, making it suitable for large-scale clinical or pharmacological toxicological detection (Kakiyama et al., 2014).

With accumulating new evidence on the involvement of bile acids in a growing number of human physiological and pathological conditions, there is renewed interest in improving the measuring techniques for various bile acids in different biological samples. Ultra-performance liquid chromatography-tandem mass spectrometry (UPLC-MS/MS) is a new method developed in the last 20 years (Sun et al., 2019; Hu et al., 2020; Lawler et al., 2020). Using the theory and principle of HPLC, UPLC has improved and innovated a number of chromatographic technologies, including small particle fillers, very low system volumes, and rapid detection methods, which increase the flux, sensitivity, and chromatographic peak capacity of analysis (Wang et al., 2015). In 2004, Waters launched the world’s first newly developed UPLC instrument (Plumb et al., 2004).

Han et al. (2015) developed an ultra-fast liquid chromatography/multireaction monitoring-mass spectrometry method for the separation and detection of 50 known bile acids. High-throughput-compatible 96-well phospholipid-depletion solid-phase extraction was adopted as a new sample preparation method. A single analysis of human serum bile acid species in the sub-nanometer concentration range has been achieved, which is far more sensitive than the previous HPLC-MS/MS. Sarafian et al. (2015) developed a 15-min UPLC method for the separation of bile acids from human biological liquid samples, requiring minimal sample preparation, for sensitive, quantitative, and targeted analysis of 145 bile acids, including primary, secondary, and tertiary bile acids. Among these, 57 bile acids were quantitatively measured using 16 marketable stability markers.

Compared with HPLC, this method has a shorter detection time, narrower peak width, lower detection limit, higher retention capacity, and higher signal-to-noise ratio. The application of UPLC technology has made a breakthrough in liquid chromatography at a higher level. Through the efforts of the majority of experimental workers in recent years, the UPLC technology has seen wide application in all walks of life, proving it to be feasible with broad application prospects.

#### Gas Chromatography-Mass Spectrometry

Gas chromatography refers to the gas mobile phase chromatographic separation method. In the early years of research, when GC/GC-MS had higher detection capabilities than liquid chromatography and HPLC, GC and GC-MS were the most commonly used bile acid detection techniques and were regarded as a reference method (Scalia, 1995). GC was first used in bile acid series analysis in 1960, and only four methyl bile acid derivatives were detected (VandenHeuvel et al., 1960).

Bile acids contain functional groups, such as carboxyl, hydroxyl, and oxo groups, and hydrogen bonds are formed between the molecules (Dutta et al., 2019). Therefore, bile acids have thermal instability and insufficient volatility for GC, and the samples need to be derived in advance. The sample needs to be fractionated in advance according to the different binders, followed by the concentration and derivation steps, which are time-consuming. The first step in the pretreatment of samples is the dissociation of conjugates, wherein alkaline hydrolysis (4 M sodium hydroxide, 115°C, pressure 15 psi) is commonly used to hydrolyze bile (Batta and Salen, 1999). Other enzymes may also be used to hydrolyze the conjugates; for example, glycine or taurine conjugates are usually hydrolyzed with a choloylglycine hydrolase, and β-glucuronidase is used to hydrolyze bile acid glucosides. The second step is transformation. The carboxyl groups in bile acids are usually converted to methyl esters, commonly using fresh diazomethane (Campbell et al., 1975). In addition, anhydrous methanol hydrochloric acid, methanol-5% sulfuric acid, or methyl alcohol in the presence of p-toluenesulfonic acid may be used. The hydroxyl groups in bile acids are often converted to trimethylsilyl ether derivatives, usually using trimethylsilanylation agents, such as *N*,*O*-bis(trimethylsilyl)acetamide and bis (trimethylsilyl)trifluoroacetamide. Oxo functional groups are susceptible to strict alkaline hydrolysis; therefore, in the presence of bile acids containing Oxo functional groups, enzymes are usually used to hydrolyze conjugated bile acids, which can be detected without oxo functional group conversion or by *o*-methyloxime or dimethylhydrazone conversion.

In order to prevent errors caused by sample pretreatment, it is necessary to select an appropriate quantitative internal standard for GC. Commonly used internal standards are coprostanol (Setchell and Matsui, 1983), nor-deoxycholic acid (Child et al., 1987), 7α,12α-dihydroxy-5β-cholan-24-oic acid (Bailey et al., 1987), Notably, bile acids labeled with stable isotopes give the most accurate results (Sjövall et al., 1985).

One of the drawbacks of GC and GC-MS is that they are very time-consuming in sample preparation, which involves extraction, purification, hydrolysis, and derivatization, as well as the inaccurate identification of stereoisomerization forms of bile acids with a single gas column, which limits the wide application of GC/GC-MS. The development of improved separation procedures and their automation will increase the analytical potential of capillary gas chromatography separation capabilities. Compared with the latest electrospray HPLC-MS, traditional GC and GC-MS have a lower sensitivity, and as a result, they have been replaced.

### Capillary Electrophoresis (Capillary Micellar Electrokinetic Chromatography)

Capillary electrophoresis for the determination of bile acid concentration is a new technique that appeared at the end of the last century, but it is not commonly used in bile acid detection. CE is a new type of liquid phase separation technology with a capillary as the separation channel and a high-voltage DC electric field as the driving force. CE consists of electrophoresis, chromatography, and their cross content. The traditional pressure driving force of chromatography is changed to a voltage driving force. Capillary isokinetic electrophoresis and micellar electrokinetic capillary chromatography are commonly used for bile acid detection. Conductance detectors and indirect UV absorption detectors are commonly used. Dimethylcyclodextrin (D-β-CD) and trimethylcyclodextrin (T-β-CD) are often added to the buffer solution to improve the selectivity of the analysis.

When the technology was first developed, it could only detect pure bile acid samples or impurities in pure bile acid drugs. For example, UDCA plays an important role in the treatment of cholesterol calculi. Quaglia et al. (1997) detected UDCA and its common impurities using high-performance capillary zone electrophoresis (UPCE) combined with indirect UV absorption detection. Yang et al. (2011) used capillary electrophoresis frontal analysis (CEFA) to study the effects of four different bile salts, cholate, deoxycholate, taurocholate, and monoketone cholate, on membrane binding of the cationic model drug, proderol.

In recent years, this technique has been applied for the detection of biological samples. Reijenga et al. (1983) used isokinetic electrophoresis combined with a conductivity detector and indirect UV absorption detector in a non-aqueous solvent (95% methanol) to detect the amounts of various conjugated bile acids in human bile. Yarabe et al. (1998) optimized the indirect photometric method of capillary zone electrophoresis (CZE) to detect the concentrations of 15 common bile acids in human plasma samples for the first time. The addition of γ-cyclodextrin to the running electrolyte significantly improved the peak resolution; however, the sensitivity of bile acid detection required further improvement.

Capillary electrophoresis has good separation capacity and detection speed, but it is not as sophisticated as HPLC. Moreover, these procedures are technically demanding and unreliable and therefore not suitable for routine analysis of biological samples (Scalia, 1995). In the future, with the improvement in detection technology, CE may be comparable to traditional HPLC.

## Bile Acid-Related Diseases

Bile acid synthesis has extensive inter-individual variation; for example, it is lower in women than in men, and is positively correlated with serum triglyceride levels (Galman et al., 2011). In addition to the individual differences in bile acids, various diseases, such as hepatobiliary, gastrointestinal, metabolic, and nervous system diseases, can also cause changes in the amount or type of bile acid in the human body. Bile acids are produced by the liver and are involved in the metabolism of intestinal microorganisms in the gut. New evidence suggests that the intestinal microflora are closely related to the risk, development, and progression of gastrointestinal cancer and hepatocellular carcinoma (Jia et al., 2018; Lee et al., 2020). Therefore, it is of great significance to understand the interconnection between the liver, bile acids, and digestive microflora. Using the relevant techniques mentioned in the previous sections, we can monitor changes in metabolism and diagnose or predict the occurrence of related diseases.

### Gastrointestinal Diseases

The intestinal tract is the main site where bile acids promote the digestion and absorption of lipids. Secondary bile acids are synthesized by intestinal flora via deconjugation, dehydrogenation, and dehydroxylation. Therefore, gastrointestinal diseases, such as inflammatory bowel disease (IBD) and gastric cancer, are closely related to bile acid metabolism in the body.

Inflammatory bowel disease is an idiopathic inflammatory bowel disease involving the ileum, rectum, and colon. Clinical manifestations include diarrhea, abdominal pain, and even bloody stools. The disease includes ulcerative colitis (UC) and Crohn’s disease (CD). Gut microbes metabolize bile acids, and because of the ecological dysregulation that occurs in IBD, bile acid metabolism is disrupted. Duboc et al. (2013) found that fecal conjugated bile acid levels were significantly elevated and secondary bile acid levels were significantly reduced in active IBD. Interestingly, patients with active IBD had higher levels of 3-OH-sulphated bile acids in their feces. Microbial deconjugation, transformation, and desulfurization activities in patients with IBD are impaired.

Gastric cancer is a malignant tumor originating from the gastric mucosal epithelium, and the vast majority of gastric cancers are adenocarcinomas. Gastric cancer has no noticeable symptoms in the early stage or has non-specific symptoms, such as upper abdominal discomfort and belching, which are often similar to the symptoms of chronic gastric diseases, such as gastritis and gastric ulcer, and are easily ignored. Therefore, the rate of early diagnosis of gastric cancer is still low (Tan, 2019). In the western world, it is most often diagnosed at an advanced stage, after distant metastasis (Digklia and Wagner, 2016). Lee et al. (2020) successfully analyzed 70 human gastric juice samples from patients with chronic superficial gastritis, intestinal metaplasia, and gastric cancer, and studied the correlation between bile acid metabolism and gastric cancer. According to the progression of chronic superficial gastritis to intestinal metaplasia and gastric cancer, there were statistical differences in the metabolism of CA and DCA. Therefore, the progression of gastric cancer may be related to changes in the composition of the intestinal microbiome. This study provides insights into the etiological mechanisms of gastric cancer progression and biomarkers for the diagnosis and treatment of gastric cancer.

### Hepatobiliary Diseases

Bile acids are produced in the liver, stored in the gallbladder, and serve a function in the gut; only a small fraction enters the blood circulation. In hepatobiliary disease, the bile acid levels in the serum vary greatly. The membrane bile acid receptor TGR5 and the FXR of the nuclear hormone receptor FXR-α are not only regulatory factors for the synthesis and transport of bile acids, but also the transcription factors and the protection sensors that are regulated by the bile acids, which induce protective cell reactions of the liver and gastrointestinal tissues, regulating inflammation, immune response, and liver regeneration (Jia et al., 2018). Thus, the metabolic changes in the liver can be reflected in the changes in bile acid contents and types, and the detection of bile acid changes can be used for the diagnosis of hepatobiliary diseases.

Bile deposition is a pathological physiological process caused by the secretion of bile and excretion disorder. It is characterized by an excessive accumulation of bile (in the liver and body circulation), cholesterol, and bilirubin, which can cause damage to liver cells and the body; moreover, long-term persistent bile silting is caused by liver fibrosis and even cirrhosis (Li et al., 2017). Bile flow damage causes bile deposition, which is characterized by elevated bile acid levels in the liver and serum, followed by liver cells, and biliary damage. The amide proton region of ^1^H MR in human bile plays an important role in distinguishing cholestasis patterns from normal ones. Ijare et al. (2009) used this method to obtain bile from normal bile ducts containing bile acids with taurine and glycine conjugates, CA, CDCA, and DCA. A lack of glycine-bound CDCA was observed in bile samples from patients with primary sclerosing cholangitis.

Intrahepatic cholestasis of pregnancy (ICP) is a special complication that occurs in the second and third trimesters of pregnancy. It is characterized by itchy skin and elevated levels of bile acid. It mainly harms the fetus and increases the morbidity and mortality of perinatal children. ICP diagnosis involves a liver anomaly examination. Its etiology is unknown and it will get better during childbirth. Liver examination includes measurement of liver transaminase, bilirubin, or bile acid levels. Tribe et al. (2010) identified changes in bile acid spectra during normal pregnancy and pregnancy with intrahepatic cholestasis and pruritus and found that ICP was associated with a significant increase in taurine- and glycine-conjugated cholic acid levels after 24 weeks of gestation; ursodeoxycholic acid (UDCA) therapy significantly reduced serum taurocholic acid and taurodeoxycholic acid concentrations. In addition to the serum bile acid test, urobile acids can also be used to detect or exclude ICP. Huang et al. (2007) tested this assay and found that urine bile acid sulfate (UBAS) ratio from UBASTEC-AUTO could distinguish patients with ICP from healthy pregnancies. At the same level of sensitivity, urine bile acid sulfate has a higher specificity than non-sulfuric urine bile acids. Nevertheless, Manzotti et al. (2019) believe that serum TBA, serum bile acid spectrum, or both, for the diagnosis of ICP is overestimated as the overall risk of bias is high; additionally, there are concerns about the applicability of the results in clinical practice, making it difficult to make recommendations and draw final conclusions based on available literature.

Urine and serum metabolomic analyses revealed bile acids as potential biomarkers for primary biliary cirrhosis (Kakiyama et al., 2013). Tang et al. (2015), using the ultra-high performance liquid chromatography-quadrupole time-of-flight mass spectrometry (UPLC/Q-TOF MS) method, showed that bile acid levels increased with the progression of primary biliary cirrhosis, and carnitine levels, such as that of propionate carnitine and butyl carnitine, decreased with the progression of primary biliary cirrhosis. Thus, they could be used to diagnose the onset and progression of primary biliary cirrhosis.

### Metabolic Disease

Low-grade tissue inflammation induced by obesity can lead to insulin resistance, which is a key cause of type 2 diabetes. Cells of the innate immune system produce cytokines and other factors that impair insulin signaling, which contributes to the link between obesity and the development of type 2 diabetes (Lackey and Olefsky, 2016). As signaling molecules, bile acids are involved in the regulation of energy metabolism and immune response *in vivo*. Therefore, when obesity or type 2 diabetes occurs, the bile acid pool in the body undergoes corresponding changes. TBA concentrations were elevated in obese patients and correlated with body mass index, but not with type 2 diabetes. In patients with type 2 diabetes, both fasting and post-prandial systemic TBA concentrations were found to be increased. However, different qualitative components of bile acids have been reported in different studies. Wu et al. (2015) found elevated levels of nine metabolites in the serum of patients with type 2 diabetes, three of which were bile acids—taurochenodeoxycholic acid, 12α-hydroxy-3-oxocholadienic acid, and glycocholic acid. Wewalka et al. (2014) found that fasting serum taurine-conjugated bile acid concentrations were higher in patients with type 2 diabetes than in those with normal glucose tolerance and were not associated with the intensification of insulin levels, but with fasting and post-load glucose. Interestingly, patients with insulin resistance, but not type 2 diabetes, showed an increased 12α-hydroxylated:non-12α-hydroxylated bile acid ratio (Chavez-Talavera et al., 2017).

Zheng et al. (2021) studied the association of HCA with obesity and diabetes. They found a strong association between metabolic disorders and HCA. The serum concentration of HCA in obese or diabetic patients was lower than that in healthy adults, and the serum HCA level was increased in post-Sleeve Gastrectomy (SG) patients. They further demonstrated that low levels of HCA were found in the feces of prediabetic patients and that increased concentrations of HCA after SG predicted remission 2 years after diabetes, thus demonstrating the feasibility of using HCA to assess the risk of future metabolic diseases.

Bile acid levels also change after metabolic surgery. Roux-en-Y gastric bypass (RYGB) surgery can alleviate type 2 diabetes; however, the mechanism of action is not fully understood. Gerhard et al. (2013) found that after Roux-en-Y Gastric Bypass (RYGB) surgery, FGF19 and bile acid levels (especially that of cholic and deoxycholic acids) were highly increased in diabetes-RYGB patients than in non-diabetic or diabetes-non-RYGB patients, suggesting that the FGF19-CYP7A1-BA pathway plays a role in the remission of type 2 diabetes after RYGB surgery. Moreover, changes in serum bile acid content can also be used to monitor the therapeutic effect of type 2 diabetes drugs. For example, after colesevelam treatment, CA synthesis was enhanced, but CDCA and DCA levels decreased in patients with type 2 diabetes. Interestingly, the total content of hydrophobic bile acids decreased, and the total bile acid pool remained stable (Brufau et al., 2010).

Gestational diabetes is defined as diabetes with normal glucose metabolism before pregnancy or diabetes with underlying glucose tolerance that does not appear until or is diagnosed during pregnancy. Gao et al. (2016) found that the levels of eight bile acids, including two dihydroxy conjugated, one trihydroxy unconjugated, and five sulfated bile acids, were significantly increased in the serum of patients with gestational diabetes compared with the control group. β-Muricholic acid (β-MCA) and dihydroxy conjugated bile acids were identified as the most suitable metabolic markers for the diagnosis and differential diagnosis, respectively, of gestational diabetes.

### Cardiovascular Disease

Metabolic syndrome is an increasingly worldwide public health problem. It refers to the pathological state of metabolic disorder of proteins, fats, carbohydrates and other substances in human body. It is a group of complex metabolic disorder syndrome. Metabolic syndrome is a constellation of five clinical symptoms, hypertension, hyperglycemia, hypertriglyceridemia, insulin resistance, and obesity (Porez et al., 2012; Spinelli et al., 2016). In addition to metabolic diseases such as diabetes mentioned above, cardiovascular diseases are also an important component. Pushpass et al. (2021) suggest that bile acids may be the link between gut microbiome and cardiovascular health. Although results from human studies have been inconsistent, there is growing evidence that these dietary components are associated with improvements in lipid cardiovascular disease risk markers, which may be related to the regulatory role of gut microbiota and bile acid metabolism. These include an increase in newly synthesized bile acids due to bile chelation, the metabolic activity of bile salts, and short-chain fatty acids produced by bacterial fermentation of fibers. Desai et al. (2017) propose that the decreased expression of Pgc1α is associated with metabolic dysfunction in cholecystitis, and that lowering serum bile acid concentration helps to inhibit cardiac metabolic and pathological changes.

### Nervous System Disease

Recent studies have shown that bile acid signaling plays a broad role in the central nervous system. Some bile acids, such as taurine DCA and UDCA, have shown neuroprotective potential in a number of experimental animal models and clinical studies on neurological diseases (Grant and DeMorrow, 2020). Recent epidemiological and molecular studies have shown that disruption of cholesterol homeostasis is associated with an increased risk of Alzheimer’s disease. In addition, brain cholesterol accumulation contributes to the progression of hepatic encephalopathy through the role of bile acid-mediated FXR. Targeting the gut microbiome–bile acid–brain axis may be a novel strategy against Alzheimer’s disease and hepatic encephalopathy. However, further research on this axis is still needed to fully understand and treat Alzheimer’s disease and hepatic encephalopathy (Jia et al., 2020).

## Conclusion and Perspectives

In summary, there are a number of bile acid detection methods; LC-MS and its derivatives are used as the mainstream techniques in laboratory research, while enzyme analysis for the detection of TBA is also being used in clinical diagnosis. Each of these methods has its own advantages and disadvantages, and the applicable conditions are different. The relationship between disease and bile acid metabolism has also becoming clearer with progress in research; however, there is still much to be learned. In terms of scientific research and technology, whether there are some properties of bile acids that have not been used in the existing detection method or whether emerging technologies can be used in the detection of bile acids needs to be addressed, in clinical diagnosis, studies are required to improve the sensitivity and specificity of these techniques, making them suitable for application in the diagnosis, identification, and even prediction of diseases. Such techniques when applied to the clinic will benefit humanity, with far-reaching significance, and further studies are needed to explore their potential.

## Author Contributions

XZ was involved in data collection and writing of the manuscript. ZL and FS were involved in conception, design, and coordination of the study. LY and GY were involved in picture and table formatting. KW supervised the project and revised the manuscript. All authors have critically reviewed the manuscript and have approved the publication of this final version of the manuscript.

## Conflict of Interest

The authors declare that the research was conducted in the absence of any commercial or financial relationships that could be construed as a potential conflict of interest.

## Publisher’s Note

All claims expressed in this article are solely those of the authors and do not necessarily represent those of their affiliated organizations, or those of the publisher, the editors and the reviewers. Any product that may be evaluated in this article, or claim that may be made by its manufacturer, is not guaranteed or endorsed by the publisher.
